# Free flap selection strategies and advances in facial soft tissue defect reconstruction

**DOI:** 10.3389/fsurg.2026.1912392

**Published:** 2026-10-09

**Authors:** Junzhi Zhao, Xing He, Kangcha Chen, Jianhui Zhou, Guoqiang Li, Jialu Liu

**Affiliations:** Department of Orthopedics (Area 3), Xiaolan Clinical Institute of Shantou University Medical College, Zhongshan, China

**Keywords:** digital surgery, facial reconstruction, facial soft tissue defects, free flaps, microsurgery, patient-reported outcomes, perforator flaps, postoperative monitoring

## Abstract

**Background:**

Reconstruction of facial soft tissue defects remains a demanding task in plastic, reconstructive, and head and neck surgery because the face combines aesthetic units with essential functions such as oral competence, speech, swallowing, breathing, eyelid protection, and facial expression. The severity, depth, and composition of the defect are the primary determinants of flap selection, especially when defects extend beyond skin and subcutaneous tissue to expose the oral cavity, pharynx, nasal cavity, skull base, or critical structures.

**Methods:**

This narrative review summarizes evidence and technical progress regarding free flap selection for facial soft tissue reconstruction. Literature was identified through PubMed, Web of Science, and Google Scholar using combinations of the terms “facial soft tissue defect,” “free flap,” “perforator flap,” and “virtual surgical planning.” Priority was given to systematic reviews, meta-analyses, clinical series, and studies published between 2019 and 2026, while classic microsurgical reports were retained when foundational.

**Results:**

Free flap choice should be guided by an integrative three-dimensional matrix: defect characteristics×patient profile×institutional resources. The anterolateral thigh flap remains a workhorse for moderate to large defects because of its thickness, long pedicle, availability of fascia lata, and nerve harvest. The radial forearm free flap remains valuable for thin and pliable resurfacing of the lips, eyelids, nasal lining, and oral lining. The deep inferior epigastric perforator flap is useful for large-volume contour restoration. Additional soft tissue options—including the lateral arm, parascapular, and medial sural artery perforator flaps—should be considered when color match, donor-site morbidity, or body habitus makes traditional workhorse flaps suboptimal.

**Conclusions:**

Facial soft tissue reconstruction requires not only flap survival but also restoration of contour, sensation, oral competence, facial animation, donor-site acceptability, and patient-reported quality of life. Mature technologies such as CTA, selective ultrasound, virtual surgical planning, intraoperative indocyanine green angiography, and structured postoperative monitoring should be distinguished from evolving tools such as augmented reality, artificial intelligence decision support, robotic-assisted anastomosis, artificial vascular conduits, and allogeneic or xenogeneic transplantation. Future research should focus on multicenter registries, standardized outcome measures, cost-effectiveness analyses, and externally validated decision-support models.

## 1 Introduction

Facial soft tissue defect reconstruction is a common and highly challenging problem in plastic surgery and head and neck surgery. These defects are primarily caused by malignant tumor resection, trauma, infection, burns, congenital malformations, and secondary deformities after previous surgery or radiotherapy (1, 2). As the most visible part of the human body, the face carries essential physiological functions, including breathing, eating, speech, swallowing, eyelid protection, and facial expression, while also serving as a major determinant of individual identity and psychological well-being (3). Patients with facial deformity frequently experience anxiety, depression, social avoidance, body-image distress, and impaired quality of life (4–6). Therefore, the goal of facial soft tissue reconstruction is not limited to wound closure and tissue coverage; it also requires restoration of facial contour, expression, oral competence, ocular protection, nasal airway patency, sensation, social confidence, and long-term quality of life (7).

The development of microsurgery has transformed facial reconstruction. Compared with local or regional flaps, free flaps provide tissue with an independent vascular supply and can be tailored to repair large, complex, or irradiated defects (1, 7). After the 1970s, the reliability of microvascular free tissue transfer gradually improved, and free flaps became indispensable for extensive facial and head and neck reconstruction. With better understanding of perforator anatomy, perforator flaps and chimeric flaps have enabled more individualized design, less donor-site morbidity, and improved contour matching (8–14). More recently, digital planning, intraoperative perfusion assessment, and structured postoperative monitoring have further improved safety and accuracy (15–18).

However, flap selection remains difficult because no single flap is ideal for all facial defects. Defects vary by anatomical subunit, surface area, depth, lining requirement, dead-space volume, exposure of cavities, functional loss, recipient-vessel availability, and previous irradiation or infection. A simple superficial cheek skin defect requires a very different reconstructive strategy from a composite defect exposing the oral cavity, nasal cavity, pharynx, skull base, or mandible. Patient factors such as diabetes, peripheral vascular disease, smoking, obesity, advanced age, previous radiotherapy, nutritional status, and donor-site preference further influence decision-making (19–22). This review therefore proposes a practical selection framework based on three linked dimensions: defect characteristics, patient profile, and institutional resources.

## 2 Core principles of free flap selection

Successful facial reconstruction begins with accurate defect assessment and appropriate selection of reconstructive tissue. Ideal free flaps should provide tissue with matching thickness, pliability, color, texture, and volume; reliable perfusion; adequate pedicle length and caliber; acceptable donor-site morbidity; and the possibility of functional reconstruction when needed (1, 9). Defect severity and depth should be placed before flap preference because underestimating defect complexity may lead to inadequate lining, insufficient volume, persistent fistula, vascular compromise, or poor functional outcomes.

### 2.1 Defect-severity-first and precise reconstruction principle

Because flap selection is multidimensional, the first step is to classify the defect according to severity, depth, and missing tissue components; this assessment should inform, rather than mechanically determine, flap selection. Simple defects involving skin and subcutaneous tissue require resurfacing with thin, pliable tissue. Defects with oral, nasal, pharyngeal, or skull-base exposure require reliable lining and separation of contaminated cavities from the external environment. Large cheek or temporal defects require volume restoration and contour support. Defects involving facial animation, lip sphincter function, tongue movement, or sensory loss require functional reconstruction.

For thin-layer skin or lining defects in delicate areas such as the eyelids, lips, nasal ala, nasal lining, and oral lining, the radial forearm free flap (RFF) remains a valuable option because it is thin, pliable, reliable, and easy to shape (19–21). RFF may include the lateral or medial antebrachial cutaneous nerve for sensory restoration, and the palmaris longus tendon can be harvested simultaneously for static sling reconstruction when lip support or facial suspension is required (22). For moderate to large soft tissue defects of the cheek, temple, perioral region, or lateral face, the anterolateral thigh flap (ALT) is commonly selected because it provides a large skin paddle, long pedicle, adjustable thickness, and low donor-site morbidity (8, 9). ALT can also be harvested as a thinned or super-thin flap for contour-sensitive reconstruction (10–12). In selected cases, the ALT flap can provide fascia lata for static suspension and may permit harvest of nerve segments for cable grafting in facial nerve reconstruction. Chimeric ALT designs can separately reconstruct external skin, intraoral lining, and dead space using independent paddles on a common vascular axis (13, 14).

For defects requiring substantial volume, such as large cheek defects, temporal hollowing, or post-radiation soft tissue atrophy, the deep inferior epigastric perforator (DIEP) flap can provide abundant soft tissue while preserving the rectus abdominis muscle (23, 24). Body habitus should be considered carefully: in obese patients, ALT and DIEP flaps may be excessively bulky and require thinning, staged debulking, or alternative thinner flaps; in very thin patients, DIEP volume may be insufficient and ALT or parascapular tissue may be more appropriate.

Additional soft tissue flaps should not be overlooked. The lateral arm free flap provides thin to moderately thin fasciocutaneous tissue, reliable vascular anatomy, acceptable color match in selected patients, and avoids sacrifice of a major hand vessel (25, 26). The parascapular flap offers a broad skin paddle, reliable anatomy, and useful contouring capacity for larger resurfacing needs (27, 28). The medial sural artery perforator (MSAP) flap supplies thin, pliable tissue with relatively concealed donor-site morbidity and may be a good alternative to RFF or ALT for selected facial or intraoral defects (29, 30). These options expand the reconstructive ladder beyond the traditional ALT–RFF–DIEP triad and allow more individualized planning. The major advantages, indications, limitations, and functional options of commonly used soft tissue free flaps are summarized in Table 1.

**Table 1:** Comparison of commonly used free flaps for facial soft tissue reconstruction.

Flap	Main advantages	Typical indications in facial soft tissue reconstruction	Limitations/donor-site considerations	Special functional options
ALT	Large skin paddle; long vascular pedicle; adaptable thickness; possible chimeric design; relatively low donor-site morbidity	Large cheek, temporal, perioral, or composite soft tissue defects requiring substantial volume	Variable perforator anatomy; may be bulky in obese patients; color mismatch may occur	Can be harvested with fascia lata for static suspension; may provide nerve graft options in selected cases
RFF	Thin, pliable, reliable; long pedicle; easy flap shaping; suitable for delicate contouring	Lip, eyelid, nasal lining, oral lining, and other thin-layer defects	Visible donor-site scar; skin graft often required; radial artery sacrifice requires vascular assessment; risk of tendon exposure and wrist contracture	Can include sensory nerves; palmaris longus tendon can be harvested for static sling reconstruction
DIEP	Large soft tissue volume; reliable perforator-based blood supply; preserves rectus abdominis muscle; favorable abdominal contouring effect	Extensive cheek defects, temporal hollowing, volume restoration after tumor resection or radiotherapy	Longer operative time; technically demanding perforator dissection; possible bulk and color mismatch	Useful for volume restoration and soft tissue contouring
Lateral arm free flap	Thin to moderately thin fasciocutaneous tissue; favorable pliability; low donor-site morbidity; no sacrifice of a major hand vessel	Selected facial or intraoral defects requiring pliable tissue and acceptable color match	Limited flap size compared with ALT; donor-site scar on upper arm; unreliable perforator vessels; relatively short pedicle; risk of radial nerve injury during dissection	Useful alternative when RFF donor-site morbidity is a concern
Parascapular flap	Broad skin paddle; good contouring capacity; reliable vascular anatomy; low failure rate in contemporary reports	Complex soft tissue defects requiring broad coverage and contour restoration	Intraoperative repositioning may be required; skin color match may vary; donor-site scar on the back	Can be designed flexibly for large soft tissue resurfacing
MSAP	Thin and pliable tissue; favorable donor-site appearance and function; useful alternative to RFF or ALT	Selected facial or oral defects requiring thin soft tissue with reduced donor-site visibility	Perforator dissection may be technically demanding; possible need for skin grafting and relatively short pedicle	Suitable when thin flap and low donor morbidity are priorities
Latissimus dorsi/TDAP	Large reliable tissue territory; muscle, musculocutaneous, or perforator-based design; long thoracodorsal pedicle	Large-volume or composite defects, dead-space obliteration, and selected through-and-through defects	Muscle bulk, seroma risk, lateral chest/back scar, possible shoulder morbidity; TDAP perforators may be variable	Muscle for dynamic or dead-space reconstruction; chimeric design
Ulnar forearm	Thin pliable tissue; alternative forearm territory; may have less conspicuous donor site in selected patients	Thin oral, lip, and facial lining defects	Ulnar artery and hand perfusion must be assessed; anatomy and skin paddle dimensions may limit use	Potential sensory nerve inclusion
PAP	Moderate soft-tissue volume; concealed posterior-thigh donor site; perforator-based design	Selected volume or contour defects when abdominal or thigh options are unsuitable	Shorter pedicle and technically demanding perforator dissection; limited direct facial evidence	Adipofascial or fasciocutaneous contour restoration

The lateral arm, parascapular, and MSAP flaps are useful alternatives when traditional workhorse flaps do not offer the best match, and their comparative features are listed in Table 1. The wider soft-tissue free-flap inventory also includes the latissimus dorsi, ulnar forearm, thoracodorsal artery perforator (TDAP), profunda artery perforator (PAP), serratus anterior, serratus fascia, and selected external pudendal artery perforator flaps. These options should be considered according to the missing tissue components, donor-site priorities, and recipient-vessel conditions; they should not be interpreted as interchangeable or as a single preferred flap (31–33).

### 2.2 Principle of minimizing donor site morbidity

The principle of minimizing donor-site morbidity requires balancing reconstructive needs against donor-site function, appearance, and patient preference. ALT donor sites are usually located in a concealed region and may be closed primarily when flap width is limited; harvest generally does not sacrifice major lower-limb motor function (34, 35). RFF provides excellent tissue quality, but its donor-site scar is visible and often requires skin grafting. Reported donor-site morbidity is higher for RFF than ALT in comparative studies and meta-analyses (36, 37). For all patients, particularly those with extremely high aesthetic requirements, the possibility of donor-site scarring, skin graft pigmentation, contour depression, sensory change, tendon exposure, and delayed healing should be fully disclosed preoperatively.

DIEP harvest preserves the rectus abdominis muscle and may improve abdominal contour, but it requires longer operative time and careful perforator dissection (23, 24). The lateral arm, parascapular, and MSAP flaps may reduce donor-site concerns in selected patients: the lateral arm flap avoids radial artery sacrifice; the parascapular donor scar is hidden on the back; and the MSAP flap can provide thin tissue with acceptable lower-leg donor morbidity (25–30). Donor-site selection should also account for occupation, clothing habits, cultural preferences, previous scars, vascular status, and willingness to accept staged revision.

### 2.3 Principle of functional reconstruction priority

Modern facial reconstruction emphasizes function as much as form. Sensory reconstruction is important for lip and oral cavity reconstruction because sensation contributes to oral competence, detection of food residue, drooling prevention, articulation, and protection from biting or burns (22). To resolve the previously inconsistent sensory recovery data, this manuscript now reports a unified range: available studies suggest that approximately 60%–80% of neurotized oral or perioral free flaps may recover protective sensation, while approximately 30%–50% may recover finer discriminatory sensation, depending on nerve coaptation method, recipient nerve availability, follow-up duration, and assessment technique (22).

Motor reconstruction is equally important in perioral defects, facial paralysis, tongue defects, and lower facial suspension. Free functioning muscle transfer, especially gracilis transfer powered by the masseteric nerve, cross-facial nerve graft, or dual innervation, can restore dynamic smile or oral competence in selected patients (38–40). Muscle-containing flaps with motor and sensory nerve components may be considered for complex tongue reconstruction, perioral sphincter deficiency, and facial paralysis, although standardized comparative evidence remains limited. Static suspension using fascia lata or palmaris longus tendon may improve resting symmetry and oral continence when dynamic reanimation is not feasible.

### 2.4 Integrative three-dimensional decision matrix

To enhance clinical utility and originality, we propose a three-dimensional matrix for flap selection: defect characteristics×patient profile×institutional resources. This matrix is deliberately not presented as a linear flap-selection algorithm because flap choice must be individualized through simultaneous clinical judgment.

1) Defect characteristics: anatomical subunit, surface area, depth, exposed cavity, need for lining, need for bulk, degree of contamination, missing muscle or nerve function, and aesthetic-unit boundaries.
2) Patient profile: age, BMI/body habitus, smoking, diabetes, peripheral vascular disease, previous radiotherapy, nutritional status, anticoagulation, occupation, hobbies, scars or tattoos, available recipient vessels, donor-site preference, ability to tolerate long surgery, and expectations regarding appearance and willingness to undergo staged revision.
3) Institutional resources: microsurgical expertise, availability of supermicrosurgery, perforator-flap experience, VSP/CTA access, ICG angiography, implantable Doppler monitoring, robotic platforms, intensive postoperative observation, emergency take-back access, salvage capability, and the quality and availability of recipient vessels.

This framework shifts flap selection from a flap-centered approach to a defect- and patient-centered approach. It is intentionally not represented by a flowchart because flap selection requires simultaneous clinical judgment regarding defect anatomy, patient preferences, recipient vessels, and institutional capability. For example, a thin lower-lip defect in a young patient may favor neurotized RFF or MSAP; a large cheek defect in a thin patient may favor ALT or parascapular flap; an obese patient requiring cheek resurfacing may benefit from a thinned ALT, lateral arm flap, or staged contouring; and a radiated, vessel-depleted neck may require longer pedicles, vein graft planning, or recipient-vessel alternatives.

## 3 Commonly used free flaps and their clinical applications

### 3.1 Anterolateral thigh flap

The ALT flap has become one of the most widely used workhorse flaps in head and neck and facial reconstruction (8, 9). Its blood supply usually arises from perforators of the descending branch of the lateral circumflex femoral artery. The pedicle is generally long and of suitable caliber for microvascular anastomosis. ALT can provide a large skin paddle and can be harvested as fasciocutaneous, adipocutaneous, myocutaneous, thinned, super-thin, or chimeric tissue according to the reconstructive need (10–14).

The ALT flap is particularly useful for cheek, temporal, perioral, and large soft tissue defects requiring volume and contour restoration. Thinned and super-thin ALT techniques have expanded its use in contour-sensitive areas, although careful preservation of subdermal and perforator perfusion is required (10–12). Chimeric ALT designs allow multiple tissue components to be inset separately, which is valuable for through-and-through cheek or perioral defects (13, 14). The ALT flap also has important functional advantages: fascia lata can be harvested for static sling reconstruction, and selected nerve branches may be used as cable grafts for facial nerve reconstruction when appropriate.

Although ALT perforator anatomy may vary, routine preoperative CTA or color Doppler ultrasound is not universally required and should not be described as mandatory. Reliable surface landmarks can be sufficient in experienced hands, and studies have shown that ALT design without preoperative Doppler or imaging can be safe (41). Handheld Doppler can be helpful but is not always accurate for perforator identification (42). Therefore, imaging should be used selectively, such as in obese patients, previously operated thighs, unclear landmarks, revision surgery, or when a super-thin or chimeric design depends on precise perforator mapping.

Color mismatch is another challenge. For selected head and neck defects, split-thickness skin grafting or staged resurfacing methods have been reported to improve skin color match and aesthetic blending, particularly when native donor skin is poorly matched (43). Secondary debulking, laser therapy, scar revision, and fat grafting may further improve contour and color transition.

### 3.2 Radial forearm free flap

The RFF remains a classic flap for thin, pliable reconstruction of the lips, eyelids, nasal lining, oral lining, buccal mucosa, floor of mouth, and other delicate facial structures (19–21). It provides a long pedicle, reliable anatomy, easy shaping, and favorable pliability. In lip reconstruction, RFF can provide both external skin and internal lining, and the intraoral portion may undergo mucosalization over time (20, 21).

The RFF is particularly useful when thinness and precise contouring are essential. Sensory nerves can be harvested with the flap and coapted to recipient nerves such as the mental or infraorbital nerve. As standardized above, protective sensation may recover in approximately 60%–80% of neurotized cases and finer discriminatory sensation in approximately 30%–50%, depending on follow-up and testing methods (22). The palmaris longus tendon may be harvested simultaneously for static sling reconstruction, which is useful for lower-lip suspension, oral competence, and selected facial paralysis cases.

The main disadvantage of RFF is donor-site morbidity, including visible scarring, need for skin grafting, tendon exposure, pigmentation, contour irregularity, sensory change, and aesthetic dissatisfaction (36, 37). Wrist contracture is an additional potential functional complication. Preoperative Allen testing, Doppler assessment, or vascular imaging should be performed when necessary to ensure adequate ulnar artery compensation. With proper screening, severe hand ischemia is rare, but the potential functional and aesthetic consequences should be discussed with every patient.

### 3.3 Deep inferior epigastric perforator flap

The DIEP flap is an important option for large-volume facial soft tissue reconstruction, particularly when major contour restoration is needed after tumor resection, trauma, or radiotherapy (23, 24). It provides abundant lower-abdominal skin and fat while preserving the rectus abdominis muscle, thereby reducing abdominal-wall morbidity compared with muscle-sacrificing flaps.

DIEP flap tissue is soft and can restore cheek fullness, temporal contour, and radiation-associated soft tissue deficiency. The donor site may provide an abdominoplasty-like contour benefit in selected patients. However, DIEP harvest is technically demanding, may prolong operative time, and abdominal skin color and texture may differ from facial skin. Fat resorption, bulkiness, and need for secondary revision should be anticipated. Preoperative CTA is more commonly used for DIEP than ALT because intramuscular perforator course and perforator selection are central to operative efficiency and safety (44–46).

### 3.4 Additional soft tissue flaps

The lateral arm, parascapular, and MSAP flaps are useful alternatives when traditional workhorse flaps do not offer the best match, and their comparative features are listed in Table 1.

The lateral arm free flap provides thin to moderately thin fasciocutaneous tissue, relatively consistent anatomy, and no sacrifice of a major hand vessel. It is useful for selected facial or intraoral defects where RFF donor-site morbidity is undesirable and ALT is too bulky (25, 26). Its limitations include smaller available surface area and a visible upper-arm scar in some patients. Unreliable perforator vessels, a relatively short pedicle, and the risk of radial nerve injury during dissection should also be considered.

The parascapular flap offers a broad skin paddle, reliable vascular anatomy, and good contouring capacity for large facial resurfacing or soft tissue coverage (27, 28). The donor scar is usually concealed on the back, and the flap can be designed flexibly. However, patient repositioning may be required, and skin color match varies.

The MSAP flap is thin, pliable, and associated with relatively favorable donor-site appearance and function. It can be considered for facial or oral defects requiring thin tissue while avoiding the visible forearm donor site (29, 30). Its main limitations are variable perforator anatomy and technically demanding perforator dissection. A possible need for skin grafting and a relatively short pedicle should also be discussed. A relatively short pedicle and possible need for skin grafting are additional limitations.

Other innovative applications should be individualized rather than applied routinely. Super-large pre-expanded flaps may help repair extensive cervicofacial defects when color and texture match are critical; multi-lobed or chimeric flaps can address three-dimensional composite defects; muscle-containing flaps with motor and sensory nerves can support tongue reconstruction or facial paralysis management; and one-stage cartilage implantation may be considered for selected ear or nasal contour reconstruction when stable soft tissue coverage is available.

## 4 Recipient-site complications, monitoring, and salvage strategies

Recipient-site complications are as important as donor-site morbidity and should be considered during flap selection and perioperative planning. Major complications include arterial thrombosis, venous thrombosis, venous congestion, partial flap necrosis, total flap loss, hematoma, infection, fistula, wound dehiscence, salivary leak, contour deformity, and delayed wound healing. Large clinical series report high overall success rates for head and neck free flaps, often above 90%–95%, but failures still occur and may cause catastrophic functional and aesthetic consequences (47–50). Contemporary head and neck free-flap series generally report total flap loss rates of approximately 2%–5%, whereas re-exploration, venous congestion, hematoma, fistula, infection, and partial necrosis vary more widely according to defect site, previous irradiation, contamination, and patient comorbidity; therefore, complication reporting should distinguish flap survival from recipient-site morbidity and functional failure (51–54, 47–50).

Risk factors for vascular crisis or flap loss include previous radiotherapy, vessel-depleted neck, diabetes, smoking, peripheral vascular disease, hypercoagulability, infection, malnutrition, long operative time, hypotension, hematoma, venous kinking, pedicle compression, and inadequate recipient-vessel selection (51–54, 47–50),. In facial reconstruction, the risk may be increased by prior tumor surgery, scarred recipient beds, salivary contamination, exposed cavities, or previous neck dissection.

Postoperative monitoring should be structured, particularly during the first 24–48 h, when most vascular crises occur; the overall planning, intraoperative assessment, and monitoring workflow is summarized in Figure 1. Clinical examination remains fundamental and includes assessment of flap color, capillary refill, turgor, temperature, bleeding on pinprick, swelling, and pain. Handheld Doppler is widely used but operator dependent. Implantable Doppler monitoring can provide continuous vascular signal monitoring and may facilitate early detection of compromise in buried flaps. However, probe displacement, wire damage, loss of coupling, signal artifact, and device malfunction may produce false alarms or an absent signal despite flap viability; these problems can lead to unnecessary imaging or an unnecessary return to the operating room. The signal should therefore be interpreted together with serial clinical examination, because a functioning signal does not exclude distal skin-paddle ischemia (55, 56). Near-infrared spectroscopy and surface temperature monitoring are additional adjuncts. Intraoperative or early postoperative indocyanine green angiography can help evaluate perfusion zones, identify marginal ischemia, and guide trimming or inset decisions (57–60).

**Figure 1:**
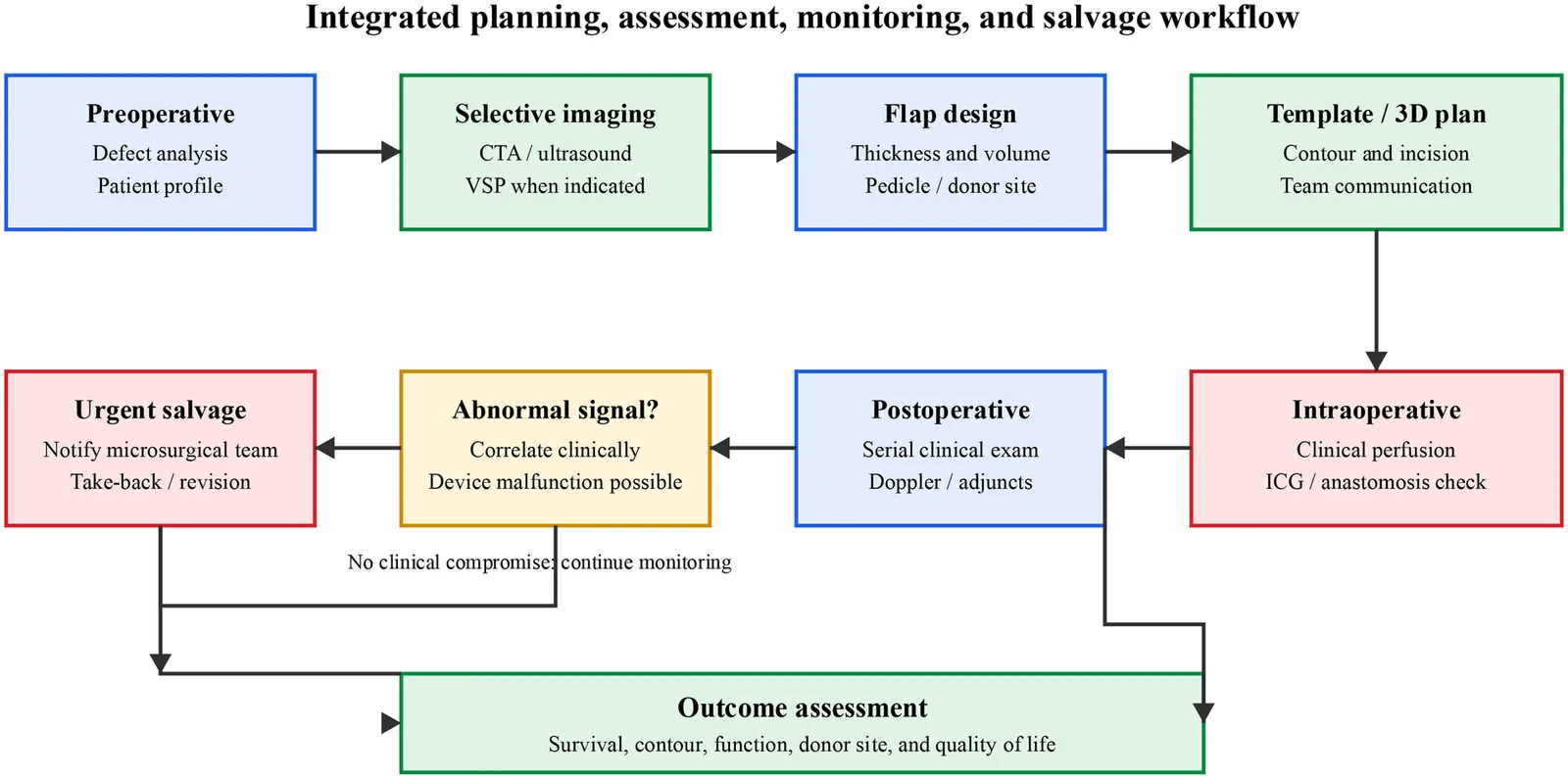
Digital planning, intraoperative assessment, and postoperative monitoring workflow. This schematic addresses perioperative planning and monitoring only; it is not a flap-selection algorithm.

Digital planning, intraoperative assessment, postoperative monitoring, and urgent salvage workflow

When vascular compromise is suspected, urgent re-exploration should not be delayed by prolonged bedside observation. Salvage protocols should include immediate notification of the microsurgical team, release of external compression, correction of systemic hypotension or hypothermia, evacuation of hematoma, assessment of pedicle kinking, revision of arterial or venous anastomosis, thrombectomy, thrombolysis in selected cases, vein grafting, or creation of an additional venous outflow. Salvage success is strongly time dependent; therefore, institutions performing facial free flap reconstruction should maintain clear monitoring pathways and emergency operating-room access.

## 5 Special clinical scenarios affecting flap selection

Special patient factors can substantially alter flap choice, surgical risk, and postoperative outcomes.

Previous radiotherapy damages recipient vessels, reduces tissue elasticity, impairs angiogenesis, and increases risks of wound dehiscence, infection, fistula, and flap-edge necrosis. In irradiated fields, well-vascularized tissue with adequate volume is preferred, recipient vessels may need to be selected outside the radiation zone, and vein grafts or alternative vessels should be planned when necessary (51, 52).

Diabetes mellitus increases risks of microvascular disease, infection, neuropathy, and delayed wound healing. Perioperative glucose control, careful recipient-vessel assessment, and avoidance of excessive tension are essential. Thin flaps with reliable perfusion and low donor-site morbidity may reduce complications in selected patients (53).

Peripheral vascular disease and smoking increase risks of thrombosis and impaired wound healing. Preoperative vascular evaluation, smoking cessation, optimization of antiplatelet or anticoagulation strategies, and selection of flaps with robust pedicles should be considered (54). RFF harvest should be avoided if hand collateral circulation is inadequate.

Advanced age is not an absolute contraindication to free flap reconstruction, but frailty, cardiopulmonary reserve, nutrition, cognitive status, and rehabilitation potential should be evaluated. Shorter operative strategies and reliable flaps may be preferred in frail patients.

Obesity and body habitus affect flap thickness, pedicle dissection, wound healing, and donor-site closure. In patients with BMI >30 kg/m^2^, ALT and DIEP flaps may be bulky, and options include thinned ALT, MSAP, lateral arm flap, staged debulking, or alternative resurfacing strategies. In very thin patients, adequate volume restoration may require ALT, parascapular tissue, stacked flaps, or secondary fat grafting.

## 6 Technical advances and future directions

Technological progress in facial soft tissue reconstruction extends beyond digital surgery. Tools should be discussed according to maturity, evidence level, cost, training requirements, and accessibility. These technologies should not be interpreted as having equivalent levels of evidence: CTA and VSP are supported mainly by systematic reviews, retrospective comparative studies, and institutional series; ICG angiography, implantable Doppler, and near-infrared spectroscopy are supported mainly by cohort studies and monitoring-focused reviews; whereas AR/MR, AI decision support, robotic-assisted microsurgery, artificial vascular conduits, tissue-engineered constructs, and xenogeneic transplantation remain supported primarily by early clinical series, scoping reviews, feasibility studies, or preclinical evidence.

### 6.1 Clinically mature and widely adopted technologies

CTA, selective ultrasound, virtual surgical planning (VSP), three-dimensional soft tissue modeling, and structured postoperative monitoring represent relatively mature technologies. CTA is most useful when perforator anatomy strongly affects surgical design, such as DIEP harvest, complex chimeric flaps, revision surgery, or unclear donor anatomy (44–46). For ALT, imaging should be selective rather than routine because experienced surgeons can use anatomical landmarks safely and handheld Doppler is imperfect (41, 42).

VSP and 3D modeling are useful for measuring defect volume, designing skin paddles, estimating soft tissue deficiency, planning incision placement, optimizing flap inset, and communicating among surgical teams. Because this review focuses on soft tissue reconstruction, the discussion is limited to soft tissue–specific applications such as contour modeling, flap volume estimation, skin-paddle design, incision planning, and flap inset planning. Cost-effectiveness varies by institution; routine use in simple defects may be difficult to justify, whereas VSP is most justified in complex, recurrent, or high-stakes defects where it may reduce operative uncertainty, operative time, secondary contouring procedures, or revision surgery (15–18).

### 6.2 Selective adjunctive technologies

Intraoperative indocyanine green angiography, implantable Doppler, near-infrared spectroscopy, and other perfusion-monitoring techniques are useful adjuncts but should complement—not replace—clinical judgment. Their value is highest in buried flaps, irradiated recipient beds, marginal perfusion zones, and complex inset. Limitations include cost, learning curve, false-positive or false-negative findings, and lack of standardized thresholds across devices (57–60).

Supermicrosurgery has expanded reconstructive possibilities by enabling anastomosis of smaller vessels and lymphatic or perforator-to-perforator reconstruction. In facial soft tissue reconstruction, it may reduce donor-site morbidity and allow more precise perforator flap transfer, but it requires advanced training, specialized instruments, and standardized curricula that are not yet universally available (61, 62).

### 6.3 Emerging and experimental technologies

Augmented reality, mixed reality, artificial intelligence decision support, robotic-assisted microsurgery, artificial vascular conduits, tissue-engineered constructs, allotransplantation, and xenotransplantation remain emerging or experimental for routine facial soft tissue reconstruction. Augmented reality may improve intraoperative visualization and image-to-patient registration, but clinical evidence remains early (17, 18). AI may assist with flap selection, risk prediction, imaging analysis, and outcome measurement, but most models lack external validation, prospective testing, interpretability, and regulatory standardization (63–66).

Robotic-assisted microsurgery may improve tremor filtration and precision in supermicrosurgical anastomosis, yet high cost, prolonged setup time, and limited access currently restrict widespread adoption (67, 68). Artificial vascular technologies and tissue-engineered conduits may eventually address vessel-depleted recipient sites, but thrombosis, infection, long-term patency, and regulatory barriers remain unresolved. Facial allotransplantation has demonstrated what is technically possible for devastating composite defects, but lifelong immunosuppression, ethical constraints, donor shortage, cost, and patient selection limit its role (69). Xenogeneic transplantation and bioengineered composite tissues remain research frontiers rather than routine clinical options.

## 7 Conclusion and outlook

Free flap transplantation remains a cornerstone of facial soft tissue defect reconstruction. The central objective is no longer flap survival alone but integrated restoration of appearance, contour, lining, sensation, oral competence, facial animation, donor-site acceptability, and quality of life. This review emphasizes that defect severity and depth should be the first determinant of flap choice and proposes an integrative matrix of defect characteristics×patient profile×institutional resources. ALT, RFF, and DIEP flaps remain essential, but lateral arm, parascapular, MSAP, chimeric, thinned, neurotized, tendon-containing, and muscle-containing flaps expand individualized options.

Several research gaps remain. First, there is a lack of randomized or well-controlled comparative studies evaluating flap types for specific facial subunits and defect severities. Second, long-term patient-reported outcome measures, aesthetic-unit scoring systems, sensory recovery tests, and donor-site satisfaction instruments are not standardized across centers. Third, AI-based prediction models, supermicrosurgery curricula, robotic-assisted techniques, and perfusion-monitoring thresholds require external validation and cost-effectiveness analysis before broad implementation.

Future work should establish multicenter prospective registries, adopt standardized patient-reported outcome measures, report defect severity using consistent classification systems, and compare reconstructive strategies by both clinical and quality-of-life outcomes. Through evidence-based flap selection, structured monitoring, and multidisciplinary collaboration, facial soft tissue reconstruction can become more precise, safer, more functional, and more patient centered.
